# Using extended complexity theory to test SMEs’ adoption of Blockchain-based loan system

**DOI:** 10.1371/journal.pone.0245964

**Published:** 2021-02-05

**Authors:** Wei Sun, Alisher Tohirovich Dedahanov, Ho Young Shin, Wei Ping Li

**Affiliations:** 1 School of Economic, Anyang Normal University, Anyang City, Henan Province, China; 2 School of Business, Yeungnam University, Gyeongsan, South Korea; 3 Railway Police College, Zhengzhou, China; Bucharest University of Economic Studies, ROMANIA

## Abstract

Blockchain-based loan system can be summed up as: information exchange between various government departments; information exchange between enterprises and various financial institutions; detection of the actual use of loans in the form of encrypted currency. This technology is supposed to reduce a lot of financing costs for SMEs on average. Therefore, this research extends complexity theory to discover the factors that affect the use of Blockchain loan systems by SMEs. Complexity, perceived risk, perceived fairness and reward sensitivity prove to have significant effects on usage intention. Complexity proves to have moderating effects on other relationships. This research may contribute to the system performance improvement and provide opportunities for SMEs to share information with financial institutions or individuals around the world, thereby providing investors with equal opportunities for competition.

## 1. Background

Small and medium-sized enterprises (SMEs) employ various forms of employment, and their mechanisms are relatively flexible, which can meet the needs of diverse forms of employment. Since the operating information of SMEs is not as transparent as that of listed companies, it brings great difficulties to financial institutions in pre-lending investigations, loan approvals, and post-lending management. The vast majority of loan work relies on manual investigation, judgment, review, and accumulation of years of work experience. Therefore, the labor cost in the entire loan process is too high. For some SMEs, in order to obtain loans faster, they may deliberately hide debts or business risks, which will lead to the failure of financial institutions’ investments. For financial institutions, since it takes a lot of time and manpower to do due diligence, they will increase loan interest rates as compensation. The above reasons create an illusion that SMEs are unwilling to borrow from financial institutions, and financial institutions are unwilling to lend to SMEs.

Blockchain-based loan system is designed to solve the problem of difficult, expensive and slow financing for SMEs. This system integrates various data and information related to SMEs such as supervision, taxation, transaction, customs, legal affairs, social security fee, and water and electricity charges. In the Blockchain-based loan system, financial institutions can query various business data of SMEs after legal authorization, and form a corporate profile, so as to judge the business status of the business before and after the loan. This system is provided and supervised by the government. SMEs who want to obtain loans quickly at low cost can voluntarily join the Blockchain-based loan system. After obtaining the consent of SMEs, relevant financial institutions can obtain their business data from the system. Moreover, since loans are issued in digital currency, the flow of funds is supervised by the financial institutions that issue loans to ensure that loans are used in actual business activities or designated purpose. The functions of this system can be summed up as: information exchange between various government departments; information exchange between enterprises and various financial institutions; detection of the actual use of loans in the form of encrypted currency. This technology is supposed to reduce a lot of financing costs for enterprises on average.

Although financial institutions vigorously promote the Blockchain-based loan system to support SMEs in financing, some SMEs hide the truly important data in the depths of the company and are unwilling to share it with financial institutions because it involves shareholder interests, trade secrets, patent privacy, transaction information, peer competition, and other specific requirements, etc. Since SMEs have different acceptance levels or some concerns about using Blockchain-based loan system, it is necessary to study the key factors that affect SMEs to carry out the system. Therefore, the focus of this research is to discover the factors that affect the use of Blockchain-based loan systems by SMEs, with the aim of enhancing usage intention and acceptance of Blockchain-based loan systems by SMEs.

## 2. Literature review

In recent years, Blockchain technology has been integrated with governments and enterprises to become a key innovation and has been widely recognized [1, 2]. Applications based on Blockchain technology are no longer limited to Bitcoin, Litecoin and other virtual currency applications. Different institutions have used Blockchain technology to try to solve practical problems that were difficult to solve in the past. Jansenn et al. (2020) [3] have introduced different factors related to Blockchain application among technics, markets and institutions. It is well known that Blockchain technology and finance are closely related, especially in terms of preventing financial fraud, privacy protection and efficiency improvement. Mohsin et al. (2019) [4] indicate that Blockchain technology can greatly improve the security of network identity verification, thereby protecting the spread of confidential information. Roriz & Pereira (2019) [5] prove that Blockchain technology can be used in the identification of vehicle insurance fraud cases in order to reduce the loss of insurance companies. In addition to these rich applications, the application of Blockchain technology in finance is truly exciting. Almasoud et al. (2020) [6] give a review about how to extend smart-contracts to reputation system based on Blochchain technology. In terms of bank innovation, Blockchain has a greater role. Wang et al. (2020) [7] make a project concerned with applying Blockchain technology to privacy management in open banking system. At the commercial level, Blockchain technology has also improved the operating efficiency of traditional e-commerce. Li et al. (2020) [8] designed a third-party logic model based on Blockchain technology to reduce the risk in e-commerce transactions. With the rapid development of financial technology, Blockchain technology has changed the original technical system at the technical level. Kabra et al. (2020) [9] propose the establishment of a Blockchain-based framework to improve the execution efficiency of financial institutions’ check clearing systems. Chang et al. (2020) [10] interviewed some financial experts to find out how to improve the quality of knowledge sharing and financial services through Blockchain technology. Sun et al. (2020) [11] test factors affecting individual investors’ switching intention who apply Blockchain-based crypto-currencies to their portfolios. From a traditional point of view, it is difficult for SMEs without credit reference, fixed assets, and stable capital flow to obtain loans. Only SMEs with good qualifications, good credit, stable business, fixed assets and stable bank flow can obtain loans from large financial institutions. Now, Blockchain technology will activate the SME loan market. With Blockchain technology, the daily activities and behaviors of SMEs will be recorded in the chain. Their business behavior, such as purchases, sales, deposits, advertising, trading activities, utility bills, tax payment will be displayed in the chain. These kinds of information can truly reflect the actual production and operation of SMEs, and provide financial institutions with great reference.

## 3. Complexity theory

Walton (2014) [12] reviews a lot of researches about application of complexity theory and suggests that this theory is consistent with new technology evaluation. Studies on variety of mechanism of application innovation based on complexity theory have been discussed over the past decade. Olya & Altinay (2016) [13] apply complexity theory in modeling tourism insurance purchase intention. Olys & Mehran (2017) [14] use complexity theory to model tourism expenditure. The researches jointly indicate that complexity theory plays an effective and important role in financial research. Complexity theory emphasizes the coordination of the relationship between financial institutions and SMEs in accordance with the principles of self-similarity, self-organization, self-learning and dynamic evolution. Financial institutions and SMEs are working together to create a Blockchain-based reputation system in a dynamic and nonlinear complex environment. When it comes to the application of this theory in business management, its advantages are even more obvious. Devereux et al. (2020) [15] explain how to achieve orientation of the corporate identity based on complexity theory. Chae (2014) [16] explain the importance of IT- enabled services’ innovation in a view of complexity theory. In addition to the above, complexity theory are also used in online medical support community [17], online shopping behavior [18], safety investigation [19], decision making [20] and leadership practice [21]. The logic of strategic thinking based on complex theory is: to formulate a financial cooperation strategy aimed at creating and improving the business ecosystem of both parties. In fact, it is the strategy of SMEs and financial institutions to strengthen their relationship management capabilities and self-adaptive capabilities under the background of the network organization structure of both parties under the strategy of a symbiotic business ecosystem. Blockchain technology provides a platform for co-creation, artificial intelligence, shared knowledge, and resource collaboration, enabling the financial cooperation between the two parties to evolve themselves. Under the effect of the adaptive mechanism, this "co-evolution" is not only reflected in the co-evolution of the lending behavior of financial institutions and SMEs, but also in the co-evolution of their cooperation strategies. The Blockchain-based lending system is designed to ultimately achieve the goal of improving the performance of SMEs and financial institutions and the success of the evolution of new business models.

Complexity may occur in the use of Blockchain-based loan system by financial institutions and SMEs. For financial institutions, although this system provides sufficient information to analyze the operating status of SMEs, the massive amount of information has brought excess workload to the managers. They need to classify the industry first, and then decide which information is important for loan review, which information is important for post-loan monitoring, and which information is only an auxiliary reference. These tasks have undoubtedly increased the complexity of the work. For SMEs, originally, many of them did not follow the standards of large enterprises to formulate work processes. Their financial process, employment system, sales channels, capital flow, cost control, etc. may not meet the requirements of financial institutions. Therefore, in order to obtain loans, small and medium-sized enterprises must adjust and upgrade their original work processes to make everything formal. And this will definitely increase the complexity of the work of SMEs. In summary, complexity is an important factor in financial institutions and SMEs in accepting Blockchain-based loan system activities.

## 4. Extending complexity theory with pull-push-mooring effects

From the perspective of motivation and behavior, SMEs choose a Blockchain-based loan system because of their inherent needs and an interest-driven performance. The decision-making behavior of SMEs is goal-oriented, and their goal is to obtain loans quickly and at low cost. When the Blockchain-based loan system cannot satisfy SMEs in some respects, SMEs will abandon it and look for alternatives instead. Therefore, this research brings together the complexity theory and pull-push-mooring effects to better study the Blockchain-based lending system.

Push factors refer to the factors that lead to dissatisfaction with existing products or services [22]. In this study, it refers to the factors that cause SMEs to be dissatisfied with Blockchain-based loan systems. Perceived risk is included in the push effect to represent factor that drive SMEs away from using Blockchain-based lending system. Pull factors refer to the factors that lead to satisfaction with new products or services [23]. In this study, it refers to the factors that cause SMEs to be satisfied with Blockchain-based loan systems. Reward sensitivity and perceived enforcement are included in pull-effects to represent factors that drive SMEs to adopt the lending system effectively. Mooring factors refer to the factors that may hinder the occurrence of transfer behavior, which generally include transfer costs, habits, subjective norms, and emotional commitments [24]. A factor named complexity is included in the mooring effects to constrain SMEs’ usage intention behavior. Pull-push-mooring effects have been applied to variety of research fields including finance, management, marketing and others.

## 5. Hypotheses and research model

### 5.1 Perceived risk (push effect)

In order to be eligible to use the Blockchain-based loan system, SMEs must improve their own information records, including but not limited to taxes, bank transfers, utility bills, wages, inventory, operating costs, etc. For SMEs, it is necessary to upgrade the current management system, train or hire specialized employees, especially to complete data that has not been recorded for many years, which brings certain risks to SMEs. Can the cost of improving this information bring the expected satisfactory low-interest loans? Will so much information uploaded to financial institutions be used for tax investigation or employment arbitration? Will senior employees who manage this information sell this information privately to peers for compensation? The most important thing is that if loans are issued in the form of digital currency, the core competitiveness of small and medium enterprises, such as suppliers, accessories and retail information, will be controlled by the government and financial institutions. In this case, the intellectual property rights of SMEs are likely to be copied at low cost. In order to apply the Blockchain-based loan system, the management system of small and medium-sized enterprises also needs to undergo many changes. Whether these changes can actually improve the company’s operating efficiency is still unknown. Perceived risk is a popular factor that has been applied to variety of research fields, such as customer loyalty [25], disaster [26], E-store image [27], hotel service [28] and online payment [29]. Based on the above discussion, it is easy to conclude that the perceived risks of SMEs include financial risks, privacy risks and performance risks. Therefore, a higher perceived risk may lead to lower intention to use Blockchain-based loan system:

H1: Perceived risk negatively affects usage intention of Blockchain-based loan system.

### 5.2 Reward sensitivity and perceived fairness (pull effects)

Blockchain-based loan system can bring fast and low-cost loans to SMEs, which is the biggest motivation for them to make changes. All small and medium-sized enterprises will conduct serious cost accounting for their investment and the returns they obtain, and strive to obtain the best returns with the lowest cost area. They will be very sensitive to returns, and if they find that companies of similar size and business have obtained loans by upgrading themselves, they will follow suit. The pursuit of rewards will prompt the central enterprise to adopt more sensitive and proactive behaviors to use Blockchain-based loan system, because reward sensitivity is closely related to the high efficiency and better completion of the work to obtain returns [30, 31] Therefore:

H2: Reward sensitivity positively affects usage intention of Blockchain-based loan system.

Perceived fairness is the most basic requirement of every industry. However, due to the complexity of financial institutions’ loan data and the difficulty of monitoring the flow of funds, it is difficult to make fair loan decisions based on business information. When various information of SME is uploaded to Blockchain-based system, financial institutions will compare the real capital needs and repayment capabilities of different companies. Commercial bribery, sales volume and cost fraud, which affect fair competition, are eliminated. In fact, because the information of many SMEs is not open and transparent, part of the loans enters the accounts of the company’s shareholders’ affiliated companies and is privately possessed by some major shareholders. In fact, sometimes the senior management of financial institutions is aware of this illegal act, but due to the exchange of benefits, it is rarely known to the outside world. This may lead to regulated business SMEs unable to obtain loans, and a large number of loan resources occupied by enterprises through commercial bribery or drawer agreements. When all relevant corporate information is displayed in the Blockchain-based loan system, managers of financial institutions who are qualified to query information will quickly discover which qualified SMEs have been treated unfairly. They can be rewarded or promoted by reporting suspicious loan behavior. Perceived fairness is so important that it exists in research in all walks of life, such as management decision [32]; Tax compliance [33]; Regulatory Policy [34]; Children’s decision-making [35]; market pricing [36]; hospitality [37]; web care [38] and engineering ethics [39]. Therefore:

H3: Perceived fairness positively affects usage intention of Blockchain-based loan system.

### 5.3 Complexity (mooring effect)

In plenty of research fields, complexity is always a key factor that affecting the adopting progress, including self-evaluation [40] and purchasing decision [41]. In order to generate real and valuable financial data; SMEs must arrange dedicated employees to engage in information entry work, which increases the complexity of the work. If the loan defaults, the SMEs’ normal capital turnover and operations will be suspended, and the travel and consumption of their top management will be affected. Even after the loan is repaid, it takes a lot of energy to restore the company’s operations to normal through technical means. Therefore, technical and operational complexity is not only a common influencing factor, but also plays a moderating role in fitting other variables to acceptance. It is a very complicated process for SMEs to change their business status to use the Blockchain-based loan system. First of all, at the technical level, SMEs need to purchase and update existing outdated equipment and hire more professional personnel to ensure that data information can be smoothly transmitted to the loan system. This process is called technological complexity. Carbonel & Rodriguez (2006) [42] indicate that the technological complexity has a moderating effect in product development. In the meantime, from the task level, since various business and financial information must be transmitted to the Blockchain-based loan system, the work content of employees must also be changed in accordance with the requirements of the Blockchain-based loan system. This increases the complexity of the task. Weiss-Cohen et al. (2018) [20] indicate that task complexity moderates decision making behavior based on experience. Also, Xu et al. (2020) [43] prove that task complexity has a moderating effect in customer service. Overly complex work may lead to the confidence of SMEs to adopt Blockchain-based loan system. Therefore:

H4: Complexity negatively affects usage intention of Blockchain-based loan system.

When the complexity increases, SMEs may worry about whether their high-paid staff can transmit operating and financial information to the system on time in accordance with the requirements of financial institutions, thereby increasing their perception of risks. The higher the complexity of routine maintenance and use of the system, the more users feel the increased risk of use, and the less willing to use the system. Therefore:

H5: Complexity will negatively moderate the relationship between perceived risk and usage intention.

Although the increase in complexity will bring some difficulties, when SMEs work hard to adapt to this complex system, they will look forward to the rewards they will obtain by using the system in the future. This is because the more they pay, the more they expect in return. When some small and medium-sized enterprises overcome the complexity, they may find themselves ahead of their peers in terms of loan efficiency, and obtain more favorable loan interest rates than their peers. Thereby, when users reduce the complexity, they may increase the intention to use the Blockchain-based loan system for more reward. Therefore:

H6: Complexity will negatively moderate the relationship between reward sensitivity and usage intention.

Accurate and complex operations make information fraud impossible. This is because dynamically updated information will make it easier for falsify data to be tracked and discovered. This is because dynamically updated information will make it easier for some people to falsify data to be tracked and discovered. Overly complex operations will put companies without professional and technical personnel at a disadvantage in the competition, thus feeling that Blockchain technology does not bring fair competition. This prompts those companies that like to pursue fairness to use the system more passively. Therefore:

H7: Complexity will negatively moderate the relationship between perceived fairness and usage intention.

**Fig 1:**
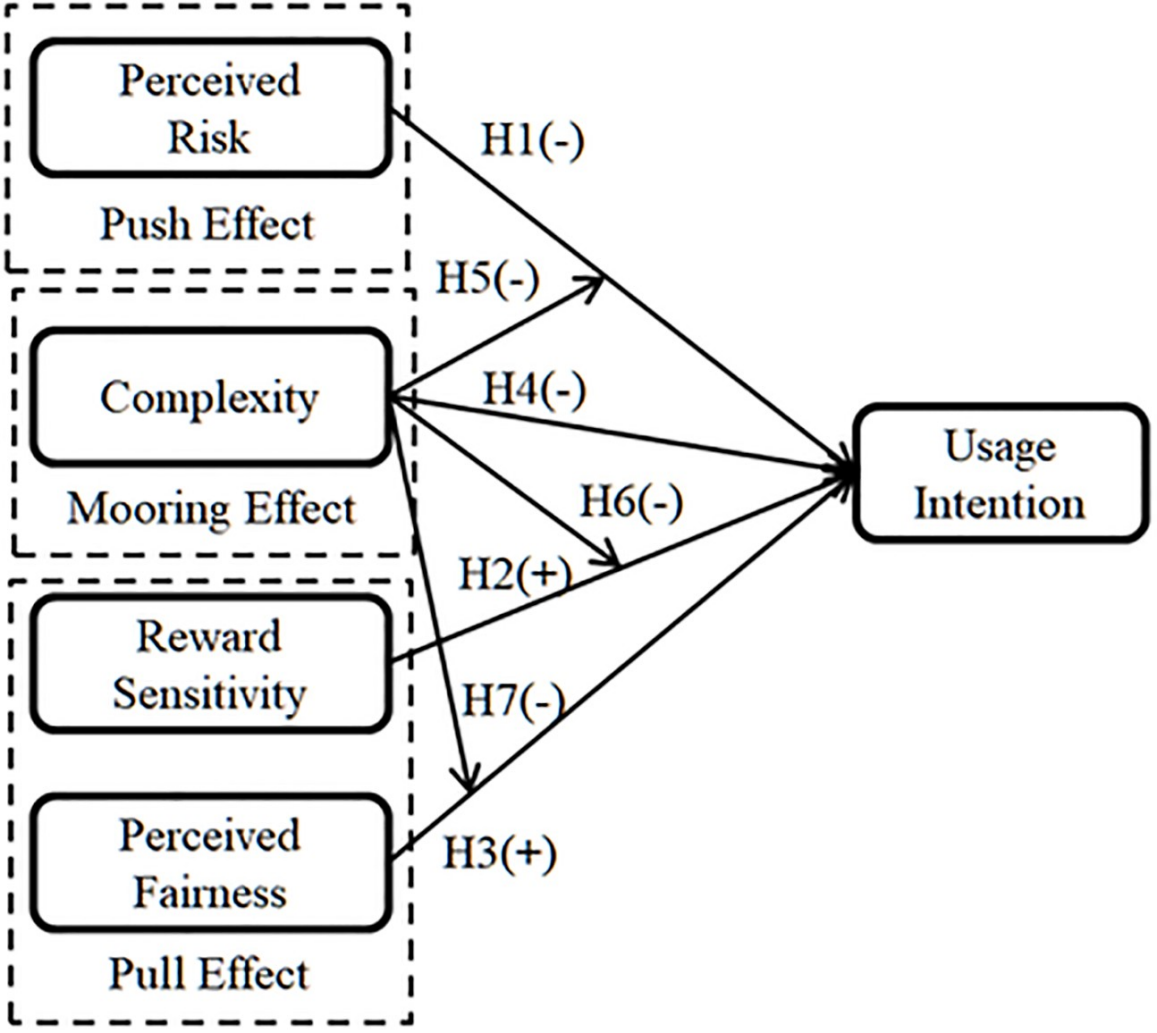
Research model.

The proposed model is showed in Fig 1.

## 6. Measurement

Questionnaire was designed to take a survey among Blockchain-based loan system users. Most of them are senior executives in finance, management, and operations industries or young heirs to family businesses in SMEs. The participants in the questionnaire are voluntary. They are fully allowed to disclose the contents of the file investigation. Most of them are under 40, have a bachelor degree or above, are willing to accept new things, and have begun to use the system to apply for loans for companies. In order to increase the reliability and validity of the document survey, we first conducted a questionnaire survey with 54 managers. Based on their feedback, we upgraded the old questionnaire and then launched a formal online questionnaire survey in April 2020. A total of 323 questionnaires were received, which is 75.5% response rate. Incomplete and invalid questionnaires were removed, leaving 296 valid questionnaires.

Perceived risk has a three items for measurement based on Wu et al. (2011) [44] study. 1. Using Blockchain-based loan system is risky. 2. It is not wise to use Blockchain-based loan system. 3. It may cost too much to get a loan by using Blockchain-based loan system.

Reward sensitivity has a three items for measurement based on Paola et al., (2015) [45] study. 1. Using Blockchain-based loan system is a good opportunity to get fast and low interest loan. 2. Using Blockchain-based loan system can quickly bring considerable returns. 3. Using Blockchain-based loan system can make our company better than peers.

Perceived fairness has a three items for measurement based on Maqsoom et al., (2019) [39] study. 1. No company can falsify data makes me feel fair. 2. Using Blockchain-based loan system gives our company more opportunities for fair competition. 3. Loans issued in the form of crypto-currency can reduce bribery and improve opportunities for fair competition.

Complexity has a three items for measurement based on Lopes et al., (2020) [41] study. 1. Using Blockchain-based loan system is a complex undertaking, with unique challenges. 2. Using Blockchain-based loan system increases the complexity of data collecting and configuration. 3. Overall, using Blockchain-based loan system has increased my workload.

Usage intention has a three items for measurement based on Xu et al., (2020) [43] study. 1. I am interested in using Blockchain-based loan system. 2. I will use Blockchain-based loan system to obtain loans for our company. 3. I will recommend Blockchain-based loan system to others to use for loan.

All the items are measured by seven-likert scale from 1 strongly disagree to 7 strongly agree. Smart-PLS was applied to this test because Blockchain-based loan system is new and PLS technics has advantages in testing new model [46, 47]. In the meantime, PLS technics can prevent restrictive distributional assumptions as well as allow modeled latent constructs by formative indicators [48]. Therefore, Smart-PLS 3.0 was chosen to test the research model. Table 1 shows a demographic statistics description, which indicates that most of the participants are under the age of 40 and have a bachelor degree or above.

**Table 1:** Demographic statistics.

Category	Subject	N	%
**Gender**	Male	203	68.7%
	Female	93	32.3%
**Education Level**	High School	34	11.6%
	Bachelor	200	67.3%
	Master	56	19.1%
	Ph.D	6	2.0%
**Age**	20–30	60	20.0%
	31–40	136	45.8%
	41–50	85	29.1%
	More than 50	15	5.1%
**Yearly Income**	< 5000$	61	20.6%
	5000–20000$	120	40.6%
	20000–40000$	103	34.8%
	>40000$	12	4.0%
**Use Frequency Of Blockchain-based Loan system per month**	None	6	2.0%
	1 time	160	54.1%
	2 or 3 times	94	31.8%
	>3 times	36	12.1%

### 6.1 Measurement model

Table 2 gives an evaluation of research model’s basic results. The values of all variables’ Cronbach’s alpha and composite reliability are all above the threshold value (0.7). The values of AVE are all above the threshold value (0.5). Standard loadings are all above the threshold (0.6). Therefore, the research model’s convergent validity and reliabilities are supported.

**Table 2:** Convergent validity, composite reliabilities testing results.

Construct	Item	Standardized Loading	AVE	Composite Reliability	Cronbach’sα
**Perceived Risk**	PR1	0.901	0.891	0.961	0.968
	PR2	0.887			
	PR3	0.865			
**Reward Sensitivity**	RS1	0.958	0.912	0.969	0.954
	RS2	0.956			
	RS3	0.951			
**Perceived Fairness**	PF1	0.935	0.901	0.964	0.956
	PF2	0.950			
	PF3	0.946			
**Complexity**	COM1	0.960	0.927	0.974	0.986
	COM2	0.964			
	COM3	0.962			
**Usage Intention**	UI1	0.946	0.893	0.962	0.980
	UI2	0.935			
	UI3	0.952			

Perceived Risk (PR), Reward Sensitivity (RS), Perceived Fairness (PR), Complexity (COM), Usage Intention (UI)

Tables 3 and 4 indicate discriminant validity test progress. Heterotrait-Monotrait Ratio (HTMT) is the ratio of between-trait and within-trait. It is the ratio of the mean value of index correlation between different dimensions to the mean value of index correlation between the same dimensions [49]. The evaluation method of HTMT is based on inferential statistics and uses confidence interval to measure discriminant validity, so it has its advantages compared with traditional tests such as cross loadings and Fornell-Larcker. All the values in Table 3 are less than the threshold value (0.85). All the values in Table 4 are less than the threshold value (1.0). Therefore, the research model’s discriminant validity is supported.

**Table 3:** Discriminant validity (Heterotrait-Monotrait Ratio).

	COM	COM*PF	COM*PR	COM*RS	PF	PR	RS	UI
COM								
COM*PF	0.187							
COM*PR	0.141	0.204						
COM*RS	0.127	0.283	0.038					
PF	0.096	0.370	0.137	0.094				
PR	0.025	0.199	0.015	0.008	0.297			
RS	0.161	0.110	0.008	0.179	0.415	0.079		
UI	0.321	0.165	0.100	0.029	0.236	0.119	0.350	

**Table 4:** Bootstrapping confidence interval up of HTMT.

COMPF-COM	0.341	PF-COMPF	0.542	PR-PF	0.471	UI-COM	0.417
COMPR-COM	0.278	PF-COMPR	0.270	RS-COM	0.266	UI-COMPF	0.291
COMPR-COMPF	0.450	PF-CPMRS	0.258	RS-COMPF	0.283	UI-COMPR	0.256
COMRS-COM	0.308	PR-COM	0.178	RS-COMPR	0.138	UI-COMRS	0.169
COMRS-COMPF	0.536	PR-COMPF	0.377	RS-COMRS	0.399	UI-PF	0.405
COMRS-COMPR	0.204	PR-COMPR	0.322	RS-PF	0.549	UI-PR	0.246
PR-COM	0.192	PR-COMRS	0.175	RS-PR	0.243	UI-RS	0.496

Perceived Risk (PR), Reward Sensitivity (RS), Perceived Fairness (PR), Complexity (COM), Usage Intention (UI)

### 6.2 Structural model

**Fig 2:**
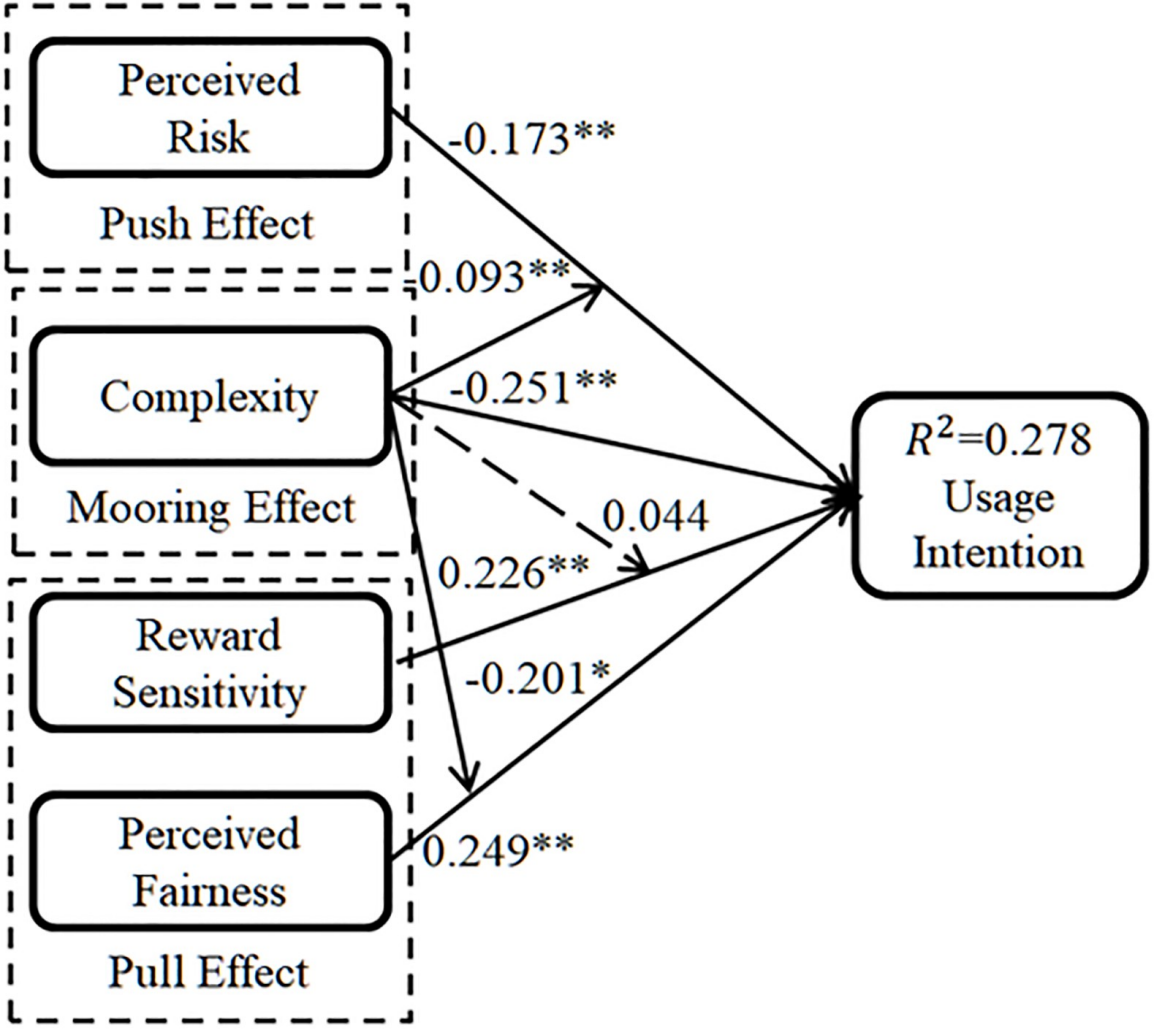
Structural model note: Standardized coefficients. ** P< 0.01; * P < 0.05.

Fig 2 illustrates the PLS analysis results including standardized path coefficient and confidence level. The path coefficient is a standardized regression coefficient that represents the direct impact of one variable on another variable. The structural mode testing results, especially the moderating tests results, are in line with previous research fields findings [50–52]. Table 5 gives the results of hypotheses testing. All the T values are above the threshold 2.58, except complexity’s moderating effect relationship between reward sensitivity and usage intention. The confidence level, which indicated by P value, is decided which hypothesis is supported. When the P is less than 0.05, the hypothesis is well supported. When P value is less than 0.01, the hypothesis is better supported. Perceived risk has a significant negative effect on usage intention with path coefficient -0.173 and P value is less than 0.01. Complexity has a significant negative effect on usage intention with path coefficient -0.251 and P value is less than 0.01. Reward sensitivity has a significant positive effect on usage intention with path coefficient 0.226 and P value is less than 0.01. Perceived fairness has a significant positive effect on usage intention with path coefficient 0.249 and P value is less than 0.01. Thus, H1, H2, H3 and H4 are supported. H5 is supported with path coefficient -0.093 and P value is less than 0.05, which indicate that complexity has a negative moderating effect on the relationship between perceived risk and usage intention. H6 is not supported with path coefficient 0.044 and P value is more than 0.05, which indicates that complexity has no moderating effect on the relationship between reward sensitivity and usage intention. H7 is supported with path coefficient -0.201 and P value is less than 0.05, which indicates that complexity has a negative moderating effect on the relationship between perceived fairness and usage intention. The R^2^ value reflects the proportion of the total variation of the dependent variable that can be explained by the independent variable through the regression relationship. Overall, usage intention is explained by the four independent variables in a large proportion of variance of 27.8%.

**Table 5:** Results of hypotheses testing.

	β	SE	T value	P	Hypotheses
PR->UI	-0.173	0.073	2.853	<0.01	Supported
COM->UI	-0.251	0.055	4.541	<0.01	Supported
RS->UI	0.226	0.070	3.216	<0.01	Supported
PF->UI	0.249	0.073	3.387	<0.01	Supported
COMPR->UI	-0.093	0.036	2.604	<0.01	Supported
COMRS->UI	0.044	0.052	0.832	>0.05	Not Supported
COM PF->UI	-0.201	0.052	2.519	<0.05	Supported

Perceived Risk (PR), Reward Sensitivity (RS), Perceived Fairness (PR), Complexity (COM), Usage Intention (UI)

**Fig 3:**
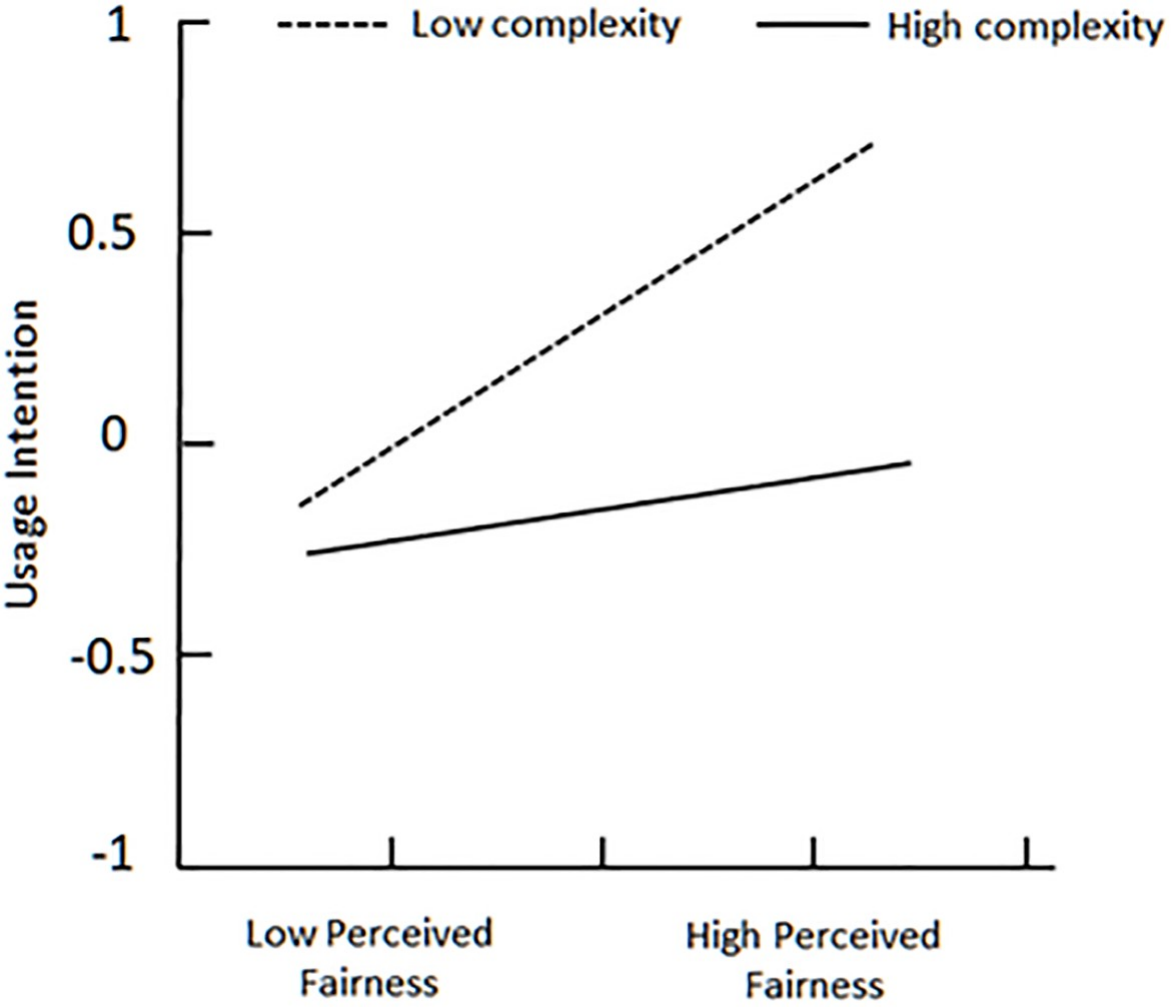
Plot of complexity’s moderating effect between perceived fairness and usage intention.

**Fig 4:**
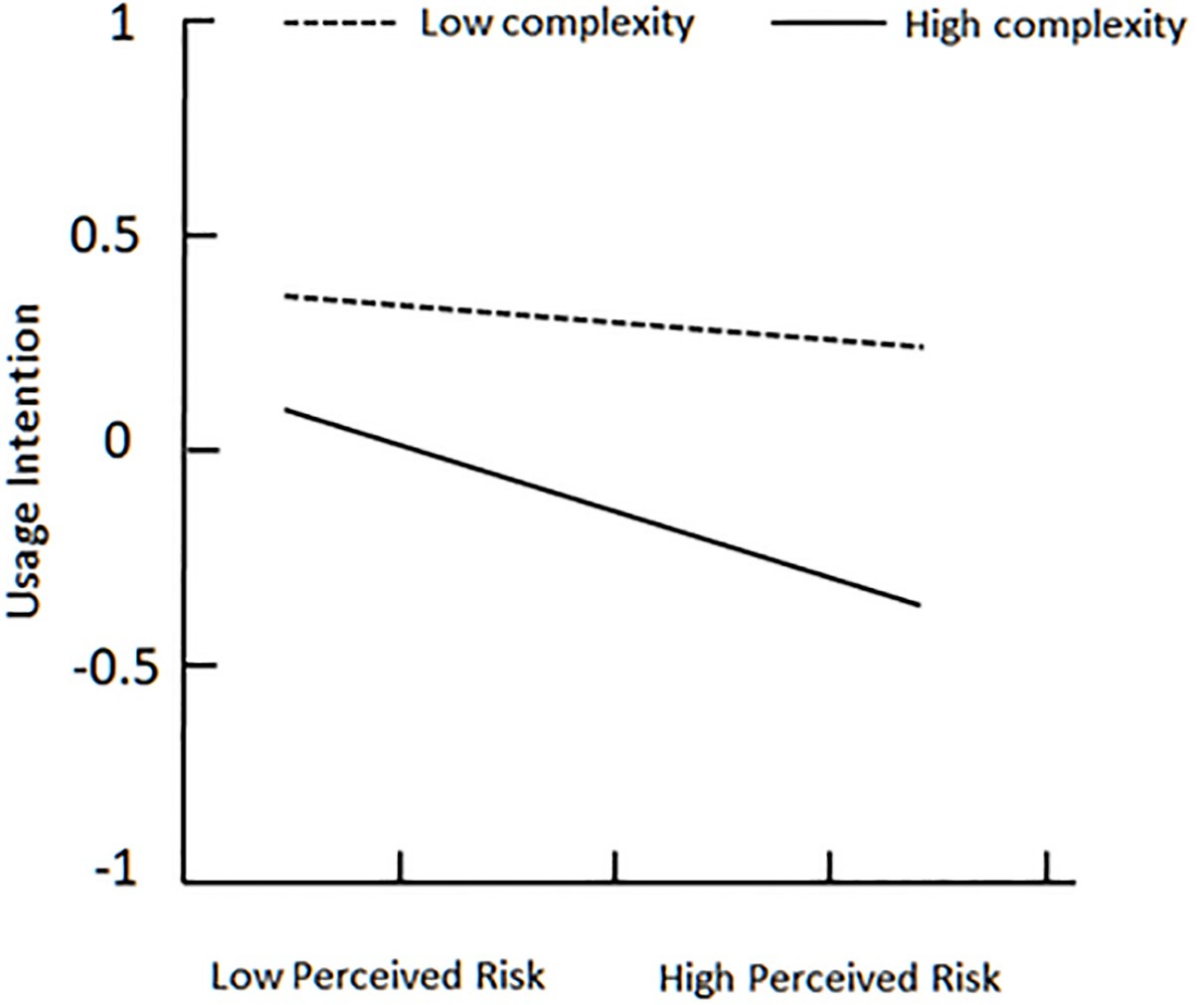
Plot of complexity’s moderating effect between perceived risk and usage intention.

**Fig 5:**
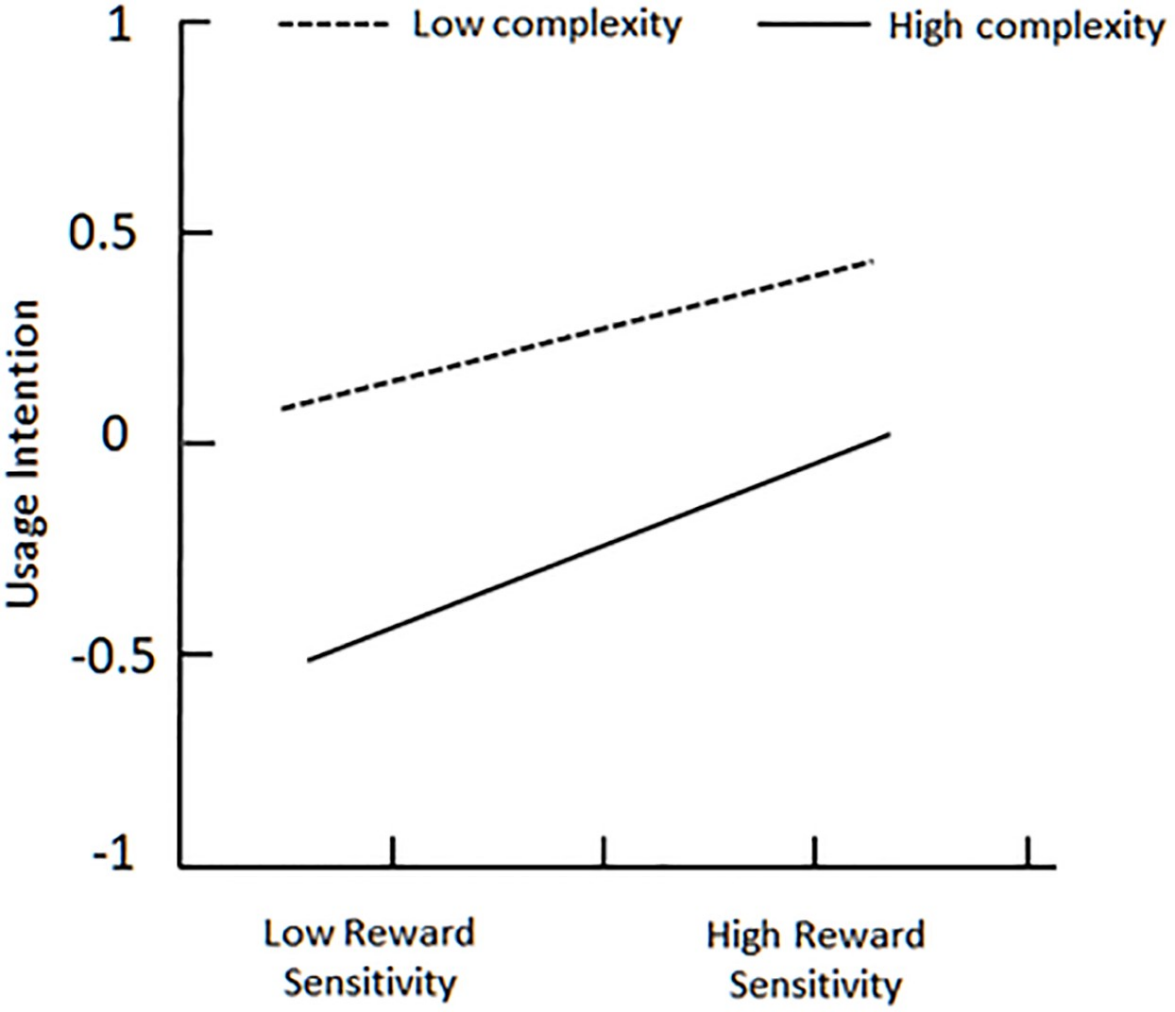
Plot of complexity’s moderating effect between reward sensitivity and usage intention.

To better explain complexity’s moderating effect between perceived fairness and usage intention, plot of two levels of complexity is showed in Fig 3. On the one hand, for users with low complexity (below the mean), perceived fairness is strongly associated with usage intention; on the other hand, the relationship is weak for users with high complexity (above the mean) [53, 54]. Fig 4 indicates that high complexity makes perceived risk more negatively associate with usage intention, when perceived risk already has a negative effect on usage intention. At the same time, low complexity makes perceived risk weakly associate with usage intention. Unfortunately, Fig 5 demonstrates that no matter complexity is high or low, reward sensitivity has almost same positive effect on usage intention.

### 6.3 Socioeconomic analysis

SMEs have shown great enthusiasm for participating in the questionnaire survey, although different companies face different difficulties, they all gave full support in this questionnaire survey. As a disadvantaged group, SMEs have shown through their enthusiastic participation that they are willing to embrace Blockchain-based loan system. All the items were given relatively high scores; even those that were originally thought to have a negative effect especially reward sensitivity and perceived fairness. To a certain extent, this shows that SMEs are looking forward to new financing channels to obtain operating funds, because the difficulty of SMEs loans has now become a widespread phenomenon restricting their development. Due to the global economic recession brought about by the epidemic and the political and economic closure of many countries around the world, the survival and development of SMEs is not optimistic. As a new financial mechanism, Blockchain-based loans may provide SMEs with opportunities for wealth fairness and they look forward to a fair competition environment based on block-chain technology.

## 7. Discussion

Complex theory is a theory widely used in various disciplines, plenty of previous researches have proved the users’ responses based on complexity related indicators contribute to adoption in different research fields [55–57]. This research uses push-pull-mooring effects to extend the complex theory to explain the SMEs’ usage intention of Blockchain-based loan system. A structural model was used to test seven hypotheses to find negative and positive effects between independent variable and dependent variable. Perceived risk was proved to have significant negative effect on usage intention [58, 59], which indicates that SMEs are willing to try this new loan system in spite of all the risks because they may believe that high risks can bring high returns. The government, as administrator of the system, should formulate strict confidentiality regulations, so that SMEs can use this system without worrying about the loss of information. At the same time, according to the principle of non-retroactivity in the law, as long as SMEs do not deliberately evade taxes, the government had better not punish those companies that actively upload business information. Otherwise, some companies will give up using the system because of risk of paying more taxes. Reward sensitivity was proved to have significant positive effect on usage intention [60, 61], which indicates that the government should reward those early enthusiastic participants and use them as role models to influence more companies to actively use the system. This system saves time for financial institutions to review materials, improves work efficiency, and reduces operating costs. Therefore, financial institutions should reward companies that use this system with lower loan interest rates. Perceived fairness proved to have a significant positive effect on usage intention [62, 63], which indicates that the government should play the role of a supervisor. On the one hand, the government can easily find unfair loan decisions made by financial institutions through the system. On the other hand, it is easy for the government and financial institutions to monitor whether the crypto-currency is actually used in the normal operation and management of the enterprise through the system, rather than being used for other purposes. Governments and financial institutions can show their fairness through clear means of rewards and punishments. SMEs that repay and pay interest on time may get preferential interest rates on their next loan.

Complexity is proved to have a significant negative effect on usage intention, which indicates that although SEMs use the system proficiently and have great stickiness to the system [64, 65], the complexity of process seems to be a burden for SEMs. The most complicated part of this system is that when SMEs use it initially, they have to enter a lot of data and standardize their company’s operations. After SMEs complete the initial standardized data input, the follow-up work is mainly simpler daily operations. Thus, the government should organize training courses on a regular basis to train representatives of SMEs so that they can better master the skills of borrowing through the system. When the company becomes more proficient in using this complex system, the company will gradually reduce the perceived risk caused by the unknown and at the same time improve a sense of fairness in using. Interestingly, complexity is proved to have no significant effect on the relationship between reward sensitivity and usage intention. This may be because when the complexity increases, users may feel that they cannot use the system efficiently and successfully, thus lowering their expectations for rewards. In this case, SMEs should reward employees who work hard to learn the Blockchain-based loan system so that they can adapt to the system more familiarly. In addition, complexity is proved to have significant negative effects on the relationship between perceived fairness and usage intention. This could be because when the complexity increases, SMEs that lack computer and financial technology talents feel that they are at an unfair disadvantage in competing with those peers with abundant talents. Therefore, SMEs should introduce talents or cultivate existing talents to enhance their competitiveness in using the new loan system. Except for this, complexity proves to have a negative moderating effect on relationship between perceived risk and usage intention. This may be because companies will possess more risk in using the new loan system with the increase of complexity. Hence, instead of training their own employees, companies can outsource or hire experts to complete the complex technical work, thereby avoiding operational risks caused by technical barriers.

These discoveries represent major advancements in technology and finance. On the one hand, this research has contributed to financial innovation by providing proof of the most influential psychological factors related to small and medium-sized enterprises lending directly through the Blockchain network system. In the past, this was a research field that academic researchers rarely paid attention to. On the other hand, this research represents an important step in the research on how SMEs’ beliefs and attitudes towards Blockchain financial technology affect the decision-making process of their use. With the support of the current comprehensive framework (PPM), our research works by incorporating complexity theory into the model and proving that this Social psychology structure plays an important role as a direct antecedent and moderating variable in the formation of system usage intentions.

## 8. Managerial implications

The results of this study show that the promotion of Blockchain-based loan system has a lot to do with the management strategies of financial institutions. It is very important to understand the psychological factors that can explain the intention of using the system, especially the role of complexity of use in this process. Encouraging SMEs to directly use Blockchain-based loan system instead of providing massive amounts of materials and multiple trips between financial institutions are very helpful to improve work efficiency. In this way, financial institutions will not only be able to fully utilize the commercial potential of Blockchain technology, but also reduce the personnel and management costs associated with the loan process.

It is important to emphasize the need to reduce the difficulty of system operation and simplify the types and quantities of materials that need to be provided. In this sense, governments and financial institutions must establish effective and friendly system operation procedures to make SMEs perceive that using this loan system is not risky and as simple, safe, fast and convenient as traditional manual channels. The interactive application of the loan system needs to optimize every detail of the loan and constrain the financial institution (for example, the loan must be completed within a limited time, and the financial institution needs special services for on-site training of users). The government and financial institutions should set up special teams and programs to train SMEs who are passionate about this system to encourage fair competition for loans.

In line with PPM theory, in order to better pull users back instead of pushing them out, financial institutions should also: 1) Improve free education on the use of information systems; 2) Reward those who are proficient and correctly using the system Enterprises, thus making this system the main way for SME loans. 3) In the first few years, if the company caused application errors due to unskilled operations, financial institutions should give understanding and try not to hold the company accountable.

The loan system based on Blockchain technology is a breakthrough achievement because it greatly shortens the negotiation time from a few weeks to a few days or hours. The government provides supporting services such as credit enhancement, interest discount, and risk compensation for small and medium enterprises using this system. By Blockchain technology, the government, enterprises and financial institutions have established mechanisms for information sharing, privacy protection and mutual trust, which not only makes corporate information true and unchangeable, but also enables financial institutions to query corporate information in accordance with laws and regulations. This can play a role in solving the financing pain points of three types of SMEs: manufacturing, foreign trade and science and technology innovation. Traditional financial institutions will carry out investment work according to their own less transparent standards, but in the future, Blockchain technology can make everyone a lender. Anyone can use their own standards to carry out small loans and track the flow of funds they have borrowed in real time. One of the main advantages of a Blockchain-based loan system is that it provides the possibility of obtaining funds from various investors and reduces the reliance on traditional financial institution processes. The system also provides opportunities for SMEs not limited to local financial institutions. They can share information with financial institutions or individuals around the world, thereby providing investors with equal opportunities for competition.

## 9. Limitations

Despite the contributions of this study, this research has some limitations. First, since the investigation is conducted in China, the administrator of the system, it could be interesting to apply this model in different countries especially in those developed countries to test the model’s consistency. Second, when the borrowers are SMEs, this model is proved to be applicable. Future research should also examine the consistency of the model when the individual acts as a borrower. Finally, most of the participants in this study are traditional companies, and their financial and business information is relatively complete. Future research should join those startups that have just been established with professional technology and patents as their main assets.

## Supporting information

S1 Appendix (DOCX)

S1 Data Description of supplementary material file: The supplementary material file is the research data of the survey. (CSV)

## References

[1] Olnes S., Ubacht J., Janssen M. (2017) Blockchain in government: Benefits and implications of distributed ledger technology for information sharing. Government Information Quarterly 34 (2017) 355–364.

[2] Frizzo B. J., Peter A., Chow W., Philippa R. A., Mentanko J., Ha D., Green S. (2020). Blockchain as a disruptive technology for business: A systematic review. International Journal of Information Management 51 (2020) 102029 10.1016/j.ijinfomgt.2019.10.014

[3] Janssen M., Weerakkody V., Ismagilova E., Sivarajah U., Irani Z. (2020). A framework for analysing blockchain technology adoption: Integrating institutional, market and technical factors. International Journal of Information Management 50, 302–309. 10.1016/j.ijinfomgt.2019.08.012

[4] Mohsin A. H., Zaidan A. A., Zaidan B. B., Albahri O. S., Albahri A. S., Alsalem M. A. (2018). Blockchain authentication of network applications: taxonomy, classification, capabilities, open challenges, motivations, recommendations and future directions. Computer Standards & Interfaces. 10.1016/j.csi.2018.12.002

[5] Roriz R., Pereira J. L. (2019). Avoiding Insurance Fraud: A Blockchain-based Solution for the Vehicle Sector Avoiding Insurance Fraud: A Blockchain-based Solution for the Vehicle Sector. Procedia Computer Science 164 (2019) 211–218. 10.1016/j.procs.2019.12.174

[6] Almasoud A. S., Hussain F. K., Hussain O. K. (2020). Smart Contracts for Blockchain-based Reputation Systems: A Systematic Literature Review. Journal of Network and Computer Applications 170 (2020) 102814 10.1016/j.jnca.2020.102814

[7] Wang H., Ma S. L., Dai H. N., Imran M., Wang T. S. (2020). Blockchain-based Data Privacy Management with Nudge Theory in Open Banking. Future Geneation Computer System 110 (2020) 812–823. 10.1016/j.future.2019.09.010

[8] Li M., Shao S. J., Ye Q. W., Xu G. Y., Huang G. Q. (2020). Blockchain-enabled logistics Finance Execution Platform for Capital-constrained E-commerce Retail. Robotics and Computer Integrated Manufacturing 65 (2020) 101962 10.1016/j.rcim.2020.101962

[9] Kabra N., Bhattacharya P., Tanwar S., Tyagi S. (2020). MudraChain: Blockchain-based framework for Automated Cheque Clearance in Financial Institutions. Future Generation Computer Systems. 102 (2020) 574–587.

[10] Chang V., Baudier P., Zhang H., Xu Q. W., Zhang J. Q., Arami M. (2020). How Blockchain can impact financial service-The overview, challenges and recommendations from expert interviewees. Technological Forecasting & Social Change 158 (2020) 120166 10.1016/j.techfore.2020.120166 32834134 PMC7306205

[11] Sun W., Dedahanov A. T., Shin H. Y., Kim K. S. (2020). Switching intention to crypto-currency market: Factors predisposing some individuals to risky investment. Plos One. 2020-06-04. 10.1371/journal.pone.0234155 32497123 PMC7272014

[12] Walton M. (2014) Applying complexity theory: A review to inform evaluation design. Evaluation and Program Planning 45 (2014) 119–126. 10.1016/j.evalprogplan.2014.04.002 24780280

[13] Olya H. G. T., Altinay L. (2016) Asymmetric modeling of intention to purchase tourism weather insurance and loyalty. Journal of Business Research 69 (2016) 2791–2800. 10.1016/j.jbusres.2015.11.015

[14] Olya H. G. T., Mehran J. (2017) Modelling tourism expenditure using complexity theory. Journal of Business Research 75 (2017) 147–158. 10.1016/j.jbusres.2017.02.015

[15] Devereux L., Melewar T. C., Dinnie K., Lange T. (2020) Corporate identity orientation and disorientation: A complexity theory perspective. Journal of Business Research 109 (2020) 413–424. 10.1016/j.jbusres.2019.09.048

[16] Chae B. (2014) A complexity theory approach to IT-enabled services (IESs) and service innovation: Business analytics as an illustration of IES. Decision Support Systems 57 (2014) 1–10. 10.1016/j.dss.2013.07.005

[17] Demjen Z. (2018) Complexity theory and conversational humour: Tracing the birth and decline of a running joke in an online cancer support community. Journal of Pragmatics 133 (2018) 93–104. 10.1016/j.pragma.2018.06.001

[18] Pappas I. O., Kourouthanassis P. E., Giannakos M. N., Lekakos G. (2017) The interplay of online shopping motivations and experiential factors on personalized e-commerce: A complexity theory approach. Telematics and Informatics 34 (2017) 730–742. 10.1016/j.tele.2016.08.021

[19] Dekker S., Cilliers P., Hofmeyr J. H. (2011) The complexity of failure: Implications of complexity theory for safety investigations. Safety Science 49 (2011) 939–945. 10.1016/j.ssci.2011.01.008

[20] Weiss-Cohen L., Konstantinidis E., Speekenbrink M., Harvey N. (2018) Task complexity moderates the influence of descriptions in decisions from experience. Cognition 170 (2018) 209–227. 10.1016/j.cognition.2017.10.005 29078094

[21] Rosenhead J., Franco L A., Grint K., Friedland. (2019) Complexity theory and leadership practice: A review, a critique, and some recommendations. The Leadership Quarterly 30 (2019) 101304 10.1016/j.leaqua.2019.07.002

[22] Jung J., Han H., Oh M. (2017). Travelers’ switching behavior in the airline industry from the perspective of the push-pull-mooring framework. Tourism Manage, Vol.59, 139–153. 10.1016/j.tourman.2016.07.018

[23] Sun W., Dedahanov A. T., Shin H. Y., Kim K. S. (2019) Regional Identity’s Role in Cambodian Microfinance Adoption: Pushing, Pulling and Mooring Factors. Asian Social Behavior. Vo.15, No.12 (2019). 10.5539/ass.v15n12p29

[24] Bansal H.S., Taylor S.F., James Y.S. (2005). Migrating to new service providers: toward a unifying framework of consumers’ switching behaviors. J. Acad. Mark. Sci. 33 (1), 96–115 10.1177/0092070304267928

[25] Marakanon L., Panjakajornsak V. (2013). Factors affecting customer loyalty of environment friendly electronics products: A conceptual model for research. Conference of the International Journal of Arts and Sciences, 6(2), 503–512.

[26] Frank B., Schvaneveldt S. J. (2016) Understanding consumer reactions to product contamination risks after national disasters: The roles of knowledge, experience, and information sources. Journal of Retailing and Consumer Services 28 (2016) 199–208. 10.1016/j.jretconser.2015.08.005

[27] Chang E. C., Tseng Y. F. (2013) Research note: E-store image, perceived value and perceived risk. Journal of Business Research 66 (2013) 864–870 10.1016/j.jbusres.2011.06.012

[28] Sun J (2014) How risky are services? An empirical investigation on the antecedents and consequences of perceived risk for hotel service. International Journal of Hospitality Management 37 (2014) 171–179. 10.1016/j.ijhm.2013.11.008

[29] Yan Q., Pang C., Liu L., Yen D. C., Tarn J. M. (2015) Exploring consumer perceived risk and trust for online payments: An empirical study in China’s younger generation. Computers in Human Behavior 50 (2015) 9–24. 10.1016/j.chb.2015.03.058

[30] Avila C., Parcet M. A., BarrósLoscertales A. (2008). A cognitive neuroscience approach to individual differences in sensitivity to reward. Neurotoxicity Research, 14(2–3), 191–203. 10.1007/BF03033810 19073426

[31] Corr P. J., DeYoung C.G., McNaughton N. (2013). Motivation and personality: A neuropsychological perspective. Social and Personality Psychology Compass, 7, pp. 158–175. 10.1111/spc3.12016

[32] Schroeder S. A., Fulton D. C. (2016): Voice, Perceived Fairness, Agency Trust, and Acceptance of Management Decisions Among Minnesota Anglers. Society & Natural Resources, 10.1080/08941920.2016.1238987

[33] Jimenez P., Iyer G. S. (2016) Tax compliance in a social setting: The influence of social norms, trust in government, and perceived fairness on taxpayer compliance. Advances in Accounting, incorporating Advances in International Accounting. 10.1016/j.adiac.2016.07.001

[34] Lind, E., Arndt, C. (2016), “Perceived Fairness and Regulatory Policy: A Behavioural Science Perspective on Government-Citizen Interactions”, OECD Regulatory Policy Working Papers, No. 6, OECD Publishing, Paris. 10.1787/1629d397-en

[35] Miller V. A., Feudtner V., Jawad A. F. (2017) Children’s Decision-Making Involvement About Research Participation: Associations With Perceived Fairness and Self-Efficacy. Journal of Empirical Research on Human Research Ethics, 1–10. 10.1177/1556264617696921 28421884 PMC5436131

[36] Lu Z., Bolton L. E., Ng S. S.L., Chen H. P. (2020) The Price of Power: How Firm’s Market Power Affects Perceived Fairness of Price Increases. Journal of Retailing. 2020 96 10.1016/j.jretai.2019.09.004

[37] Yao S., Wang X. Y., Yu H. Y., Guchait P. (2019) Effectiveness of error management training in the hospitality industry: Impact on perceived fairness and service recovery performance. International Journal of Hospitality Management 79 (2019) 78–88. 10.1016/j.ijhm.2018.12.009

[38] Raju A. (2019) Can reviewer reputation and webcare content affect perceived fairness? Journal of Research in Interactive Marketing. Vol. 13 No. 4, 2019 pp. 464–476. 10.1108/JRIM-05-2018-0065

[39] Maqsoom A., Wazir S. J., Choudhry R.M., Thaheem M. J., Zahoor H. (2020). Influence of Perceived Fairness on Contractors’ Potential to Dispute: Moderating Effect of Engineering Ethics. J. Constr. Eng. Manage., 2020, 146(1): 04019090 10.1061/(ASCE)CO.1943-7862.0001740

[40] Rosopa P. J., McIntyre A. L., Fairbanks I. N., D’Souza K. B. (2019) Core self-evaluations, job complexity, and net worth: An examination of mediating and moderating factors. Personality and Individual Differences 150 (2019) 109518 10.1016/j.paid.2019.109518

[41] Lopes E. L., Yunes L. Z., Freire O. B. L., Herrero E., Pinochet L. H. C. (2020) The role of ethical problems related to a brand in the purchasing decision process: An analysis of the moderating effect of complexity of purchase and mediation of perceived social risk. Journal of Retailing and Consumer Services 53 (2020) 101970 10.1016/j.jretconser.2019.101970

[42] Carbonell P., Rodriguez A. I. (2006) Designing teams for speedy product development: The moderating effect of technological complexity. Journal of Business Research 59 (2006) 225–232. 10.1016/j.jbusres.2005.08.002

[43] Xu YU. Z., Shieh C. H., Esch P. V., Ling I. L. (2020) AI customer service: Task complexity, problem-solving ability, and usage intention. Australasian Marketing Journal. 10.1016/j.ausmj.2020.03.005

[44] Wu P. C. S., Yeh G. Y. Y., Hsiao C. R. (2011). The effect of store image and service quality on brand image and purchase intention for private label brands. Australasian Marketing Journal. 19, 30–39. 10.1016/j.ausmj.2010.11.001

[45] Paola F. C., Avila C., Aina R. P., Noelia V. C., Bustamante J. C., Costumero V., Patricia R. N., Alfonso B. L. (2015) Reward Sensitivity Modulates Brain Activity in the Prefrontal Cortex, ACC and Striatum during Task Switching. 4 13 10.1371/journal.pone.0123073 PMC4395363 25875640

[46] Ke W., Liu H., Wei K. K., Gu J., Chen H. (2009). How do mediated and non-mediated power affect electronic supply chain management system adoption? The mediating effects of trust and institutional pressures. Decision Support Systems, 46(4), 839–851.

[47] Sun W., Dedahanov A. T., Shin H. Y., Kim K. S. (2019) Extending UTAUT Theory to Compare South Korean and Chinese Institutional Investors’ Investment Decision Behavior in Cambodia: A Risk and Asset Model. Symmetry. 2019, 11(12), 1524; 10.3390/sym11121524

[48] Goode S., Lin C., Tsai J. C., Jiang J. J. (2015). Rethinking the role of security in clien satisfaction with Software-as-a-Service (SaaS) providers. Decision Support Systems, 70, 73–85. 10.1016/j.dss.2014.12.005

[49] Hensele J., Ringle C, M., Sarstedt M. (2015). A new criterion for assessing discriminant validity in variance-based structural equation modeling. J. of the Acad. Mark. Sci. (2015) 43:115–135. 10.1007/s11747-014-0403-8

[50] Martin H S., Herrero A. (2012) Influence of the user’s psychological factors on the online purchase intention in rural tourism: Integrating innovativeness to the UTAUT framework. Tourism Management 33 (2012) 341–350. 10.1016/j.tourman.2011.04.003

[51] Bai Q. Y., Lin W. P., Wang L. (2016) Family incivility and counterproductive work behavior: A moderated mediation model of self-esteem and emotional regulation. Journal of Vocational Behavior 94 (2016) 11–19. 10.1016/j.jvb.2016.02.014

[52] Hong W., Liu R. D., Oei T. P., Zhen R., Jiang S. Y., Sheng X. T. (2019). The mediating and moderating roles of social anxiety and relatedness need satisfaction on the relationship between shyness and problematic mobile phone use among adolescents. Computers in Human Behavior 93 (2019) 301–308. 10.1016/j.chb.2018.12.020

[53] Liu Q. Q., Zhang D. J., Yang X. J., Zhang Y. C., Fan C. Y., Zhou Z. K. (2018). Perceived stress and mobile phone addiction in Chinese adolescents: A moderated mediation model. Computers in Human Behavior 87 (2018) 247–253. 10.1016/j.chb.2018.06.006

[54] Oliveira T., Martins R., Sarker S., Thomas M., Popovic A. (2019) Understanding SaaS adoption: The moderating impact of the environment context. International Journal of Information Management 49 (2019) 1–12. 10.1016/j.ijinfomgt.2019.02.009

[55] Han H., Olya H.G., Kim J., Kim W., (2018) Model of sustainable behavior: assessing cognitive, emotional and normative influence in the cruise context. Bus. Strateg. Environ. 27 (7), 789–800. 10.1002/bse.2031

[56] Brun I., Rajaobelina L., Ricard L., Berthiaume B., (2017). Impact of customer experienceon loyalty: a multichannel examination. Serv. Ind. J. 37 (5–6), 317–340. 10.1080/02642069.2017.1322959

[57] Han H., Al-Ansi A., Olya H.G., Kim W., (2019) Exploring halal-friendly destination attributes in South Korea: Perceptions and behaviors of Muslim travelers toward a non-Muslim destination. Tourism Management 71, 151–164. 10.1016/j.tourman.2018.10.010

[58] Marriott H. R., Williams M. D. (2018). Exploring consumers perceived risk and trust for mobile shopping: a theoretical framework and empirical study. Journal of Retailing and Consumer Services, 42(5), 133–146. 10.1016/j.jretconser.2018.01.017

[59] Khedmatgozar H. R., Shahnazi A. (2018). The role of dimensions of perceived risk in adoption of corporate internet banking by customers in iran. Electronic Commerce Research, 18(2), 389–412. 10.1007/s10660-017-9253-z

[60] Schreuders E., Braams B. R., Blankenstein N. E., Peper J. S, Gurolu B., Crone E. A. (2018). Contributions of reward sensitivity to ventral striatum activity across adolescence and early adulthood. Child Development. 10.1111/cdev.13056 29536503 PMC5969258

[61] Milyavskaya M., Inzlicht M., Johnson T., Larson M. J. (2018). Reward sensitivity following boredom and cognitive effort: a high-powered neurophysiological investigation. Neuropsychologia, 123 10.1016/j.neuropsychologia.2018.03.033 29601888

[62] Chong V. K., Loy C. Y., Masschelein S., Woodliff D. R. (2018). The effect of performance evaluation schemes on predicted transfer prices: do leadership tone and perceived fairness concerns matter? Management Accounting Research, 2018. 10.1016/j.mar.2018.02.003

[63] Schmidt L., Bornschein R., Maier E. (2020). The effect of privacy choice in cookie notices on consumers’ perceived fairness of frequent price changes. Psychology And Marketing, 37(9), 1263–1276. 10.1002/mar.21356

[64] Mehran J., Olya H, G. T. (2020). Canal boat tourism: Application of complexity theory. Journal of Retailing and Consumer Services 53 (2020) 101954 10.1016/j.jretconser.2019.101954

[65] Dekker S. (2013). Drifting into failure: complexity theory and the management of risk. Chaos & Complexity Theory for Management Nonlinear Dynamics. 10.4018/978-1-4666-2509-9.ch011

