# Supplementary material for: Using extended complexity theory to test SMEs’ adoption of Blockchain-based loan system
Source: PLoS One. 2021 Feb 5;16(2):e0245964. doi: 10.1371/journal.pone.0245964 (PMC7864469; doi:10.1371/journal.pone.0245964)
Supplement: S1 Appendix — (DOCX) [file pone.0245964.s001.docx]

**Appendix**

**Using Extended Complexity Theory to Test SMEs’ Adoption of Blockchain-based Loan System** (20-04-2020)

If you agree, please paste√in the bar of “I agree”

| **Disclaimer** | |
| --- | --- |
| 1. I volunteered to take part in this anonymous survey.  2. All information is authorized to be public.  3. No conflict of interest exists. | I agree______ |

Please paste√ in the bar of your choice

|  | Male | Female |
| --- | --- | --- |
| Gender |  |  |

Please paste√ in the bar of your choice

|  | High School | Bachelor | Master | Ph.D |
| --- | --- | --- | --- | --- |
| Education level |  |  |  |  |

Please paste√ in the bar of your choice

|  | 23-30 | 31-40 | 41-50 | More than 50 |
| --- | --- | --- | --- | --- |
| Age |  |  |  |  |

Please paste√ in the bar of your choice

|  | <50,000 | ≥50,000&<100,000 | ≥100,000&<200,000 | ≥200,000 |
| --- | --- | --- | --- | --- |
| Yearly Income  (us dollar) |  |  |  |  |

Please paste√ in the bar of your choice (1-strongly disagree; 7-strongly agree)

| Perceived risk | ① | ② | ③ | ④ | ⑤ | ⑥ | ⑦ |
| --- | --- | --- | --- | --- | --- | --- | --- |
| Using Blockchain-based loan system is risky. |  |  |  |  |  |  |  |
| It is not wise to use Blockchain-based loan system. |  |  |  |  |  |  |  |
| It may cost too much to get a loan by using Blockchain-based loan system. |  |  |  |  |  |  |  |

Please paste√ in the bar of your choice (1-strongly disagree; 7-strongly agree)

| Reward sensitivity | **①** | **②** | **③** | **④** | **⑤** | **⑥** | **⑦** |
| --- | --- | --- | --- | --- | --- | --- | --- |
| Using Blockchain-based loan system is a good opportunity to get fast and low interest loan. |  |  |  |  |  |  |  |
| Using Blockchain-based loan system can quickly bring considerable returns. |  |  |  |  |  |  |  |
| Using Blockchain-based loan system can make our company better than peers. |  |  |  |  |  |  |  |

Please paste√in the bar of your choice (1-strongly disagree; 7-strongly agree)

| Perceived fairness | **①** | **②** | **③** | **④** | **⑤** | **⑥** | **⑦** |
| --- | --- | --- | --- | --- | --- | --- | --- |
| No company can falsify data makes me feel fair. |  |  |  |  |  |  |  |
| Using Blockchain-based loan system gives our company more opportunities for fair competition. |  |  |  |  |  |  |  |
| Loans issued in the form of crypto-currency can reduce bribery and improve opportunities for fair competition. |  |  |  |  |  |  |  |

Please paste√in the bar of your choice (1-strongly disagree; 7-strongly agree)

| Complexity | **①** | **②** | **③** | **④** | **⑤** | **⑥** | **⑦** |
| --- | --- | --- | --- | --- | --- | --- | --- |
| Using Blockchain-based loan system is a complex undertaking, with unique challenges. |  |  |  |  |  |  |  |
| Using Blockchain-based loan system increases the complexity of data collecting and configuration. |  |  |  |  |  |  |  |
| Overall, using Blockchain-based loan system has increased my workload. |  |  |  |  |  |  |  |

Please paste√in the bar of your choice (1-strongly disagree; 7-strongly agree)

| Usage intention | **①** | **②** | **③** | **④** | **⑤** | **⑥** | **⑦** |
| --- | --- | --- | --- | --- | --- | --- | --- |
| I am interested in using Blockchain-based loan system. |  |  |  |  |  |  |  |
| I will use Blockchain-based loan system to obtain loans for our company. |  |  |  |  |  |  |  |
| I will recommend Blockchain-based loan system to others to use for loan. |  |  |  |  |  |  |  |
